# The complete chloroplast genome of *Enhalus acodoides* and its phylogenetic analysis

**DOI:** 10.1080/23802359.2019.1678430

**Published:** 2019-10-21

**Authors:** Tao Liu, Zhongjie Wu, Xuli Jia, Shiquan Chen, Zefu Cai, Jie Shen, Daoru Wang

**Affiliations:** a Hainan Academy of Ocean and Fisheries Sciences, Haikou, People's Republic of China;; b Hainan Provincial Key Laboratory of Technology for Tropical Seawater Aquaculture, Haikou, People's Republic of China;; c Laboratory of Genetics and Breeding of Marine Organism, College of Marine Life Sciences, Ocean University of China, Qingdao, People's Republic of China

**Keywords:** Seagrass, *Enhalus acodoides*, chloroplast genome, phylogenetic analysis

## Abstract

Complete chloroplast genome of *Enhalus acodoides* was obtained in this work. Circular mapping revealed that the complete chloroplast sequences of *E. acodoides* was 176,748 bp in length and had an overall GC content of 38.3%, encoded 132 genes which contained 86 protein-coding genes (PCGs), 38 transfer RNA genes (tRNA) and 8 ribosomal RNA genes (rRNA). The phylogenetic tree shows that *E. acodoides* had a closer relationship with *Thalassia hemprichii* in Hydrocharitaceae and its analysis will help better understand the evolution of Alismatales species.

Seagrasses are the only flowering plants or angiosperms inhabiting coastal and marine environments, with a worldwide distribution in temperate and tropical regions (Logan 1992; Phang 2000). They can form extensive meadows called “Seagrass beds” (Short et al. 2007) which provide shelter and food to a variety community of animals, from tiny invertebrates to large marine mammals(Nakaoka 2005; Mazarrasa et al. 2015). *Enhalus acodoides* (family Hydrocharitaceae) is one of the important flowering seagrass which was widely distributed in the tropical ocean intertidal zone of the Indian Ocean-West Pacific (Nakajima et al. 2014). It has the characteristics of morphologically large, long-lived and fast growth, which is the advantage species of coastal waters (Sakayaroj et al. 2010). But the molecular biology analysis (organelle genome.eg) of *E. acoroides* has not been reported by far.

In this study, we obtained the complete chloroplast genome of *E. acoroides.* The specimen was collected from Li’an Port, Lingshui County, Hainan Province, China (18°25′46.65″N, 110°03′3.00″E), and stored at the Culture Collection of Seaweed at the Ocean University of China with an accession number 2018030556. Total DNA was extracted using the modified CTAB method. Paired-end reads were sequenced using Illumina HiSeq × Ten system (Illumina, San Diego, CA). The complete chloroplast genomes were assembled using the programme NOVOPlasty (Dierckxsens et al. 2017). Sequence annotation was added using Geneious R10. The annotated sequence was submitted to GenBank with the accession number MN384260 and phylogenetic tree analysis of Alismatales species was carried out.

The complete chloroplast genome of *E. acoroides* is a circular DNA molecule measuring 176,748 bp in length with the overall GC content of 38.3%. The nucleotide composition was 30.79% A (54,428 bp), 19.07% G (33,710 bp), 30.87% T (54,516 bp) and 19.26% C (34,049 bp). The chloroplast genome of *E. acoroides* encoded 132 genes which contained 86 protein-coding genes (PCGs), 38 transfer RNA genes (tRNA) and 8 ribosomal RNA genes (rRNA). Among the 86 PCGs, 1 gene (psbT) uses ATC as the start codon and 5 genes (cemA, rps19, ycf1, ycf1-D2 and rps19-D2) use GTG as the start codon, the rest 80 PCGs treated the typical initiation ATG as the start codon; 39 PCGs had TAA as termination codon, 28 PCGs had TGA as termination codon and 19 PCGs had TAG as termination codon. Moreover, all tRNA genes were provided with standard cloverleaf secondary structures. There are four ribosomal RNAs (rrn4.5 rRNA, rrn5 rRNA, rrn16 rRNA and rrn23 rRNA) that are repeated in pairs.

Phylogenetic analysis was conducted using 44 shared plastid protein sequences from 14 Alismatales plastid genomes, using *L. Squamigera* (family Asparagales) as an outgroup. Concatenated alignments were generated and poorly aligned regions were removed by using the Gblocks server. The results showed that all Alismatales taxa were clearly separated according to their original class (Figure 1). The Hydrocharitaceae species formed a branch, in which *E. acoroides* showed a closer relationship with *Thalassia hemprichii.* The determination of the complete plastid genome sequence will help the understanding of Alismatales evolution.

**Figure 1.**
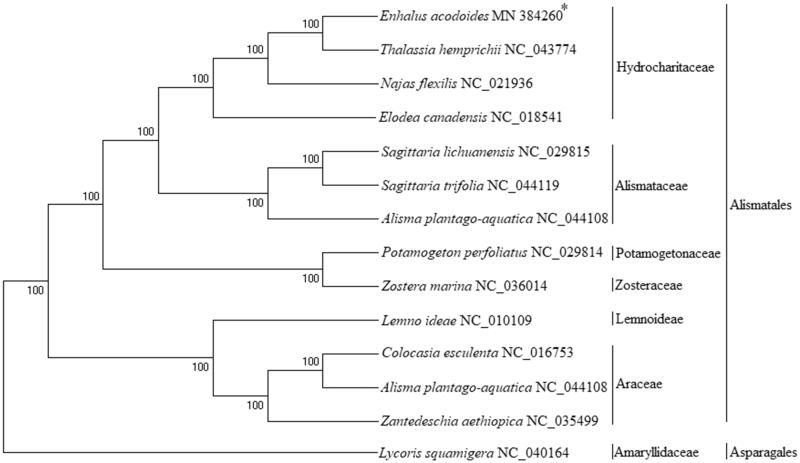
Phylogenetic tree (Bayesian method) based on the complete plastid genome of *Alismatales* species and an outgroup *Asparagales* species. The asterisks after species names indicate newly determined plastid genomes.
